# Ten Years of Euromelanoma in Hungary: Nationwide Trends and Risk Factors for Skin Cancer in Central–Eastern Europe

**DOI:** 10.3390/cancers17233749

**Published:** 2025-11-24

**Authors:** Benjamin Tamás Papp, Krisztina Toplenszky, Henriette Ócsai, Ildikó Csányi, Lajos Kemény, Rolland Gyulai, Judit Oláh, Eszter Baltas

**Affiliations:** 1 Department of Dermatology and Allergology, Albert Szent-Györgyi Medical School, University of Szeged, H-6720 Szeged, Hungary; papp.benjamin@brc.hu (B.T.P.); toplenszky.krisztina@med.u-szeged.hu (K.T.); ocsai.henriette@med.u-szeged.hu (H.Ó.); csanyi.ildiko@med.u-szeged.hu (I.C.); kemeny.lajos@med.u-szeged.hu (L.K.); gyulai.rolland.peter@szte.hu (R.G.); lazarne.olah.judit@med.u-szeged.hu (J.O.); 2 HUN-REN Biological Research Centre, Institute of Biochemistry, Synthetic and Systems Biology Unit, H-6726 Szeged, Hungary; 3 Hungarian Centre of Excellence for Molecular Medicine—Biological Research Centre (HCEMM-BRC) Systems Immunology Research Group, H-6726 Szeged, Hungary; 4 Department of Dermatology, Békés County Hospital, H-5700 Gyula, Hungary; 5 Department of Dermatology, Venereology and Oncodermatology, Faculty of Medicine, University of Pécs, H-7632 Pécs, Hungary; 6 Department of Oncotherapy, Albert Szent-Györgyi Medical School, University of Szeged, H-6720 Szeged, Hungary

**Keywords:** screening, risk factors, sunbed use, behavioral interventions, public health, skin cancer prevention, Euromelanoma

## Abstract

Background/Objectives: Large-scale, country-specific data on skin cancer predictors are scarce in Hungary. The Euromelanoma campaign offers a decade-long opportunity to investigate constitutional, behavioral, and motivational risk factors in a Central European setting through a national cross-sectional analysis. Methods: Between 2009 and 2018, Hungarian participants underwent dermatological screening. Diagnoses of clinically suspicious skin cancers were based on dermoscopic assessment, as histopathological confirmation was not systematically available. Among 18,598 standardized surveys, logistic regression identified independent predictors of clinically suspicious skin cancers overall and separately for melanoma and non-melanoma skin cancers (NMSCs). Results: Clinically suspicious skin cancers were detected in 3.9% of participants (1.7% melanoma, 2.3% NMSC). Strong predictors across all cancer types were atypical nevi (OR 4.75), personal history of NMSC (OR 3.42), and melanoma (OR 1.99). For melanoma, atypical nevi (OR 13.12), prior melanoma (OR 5.95), heavy sunbed use (OR 2.15), and trunk lentigines (OR 1.47) were significant. For NMSC, age (OR 1.08 per year), personal history of NMSC (OR 4.75), family history of melanoma (OR 2.41), and atypical nevi (OR 1.76) were dominant. Screening motivation influenced detection: participants attending for a changing lesion had higher odds of suspicious findings, whereas those attending for routine checks, family/friend history, or “many moles” had lower odds. Conclusions: Over a decade of Euromelanoma screening, atypical nevi, prior skin cancer history, and heavy sunbed use emerged as the strongest predictors of suspicious skin cancers. Participant motivation shaped detection patterns, supporting risk-stratified screening, targeted public education, and stricter regulation of artificial ultraviolet exposure.

## Simple Summary

Hungary has participated in the Euromelanoma campaign since 2009, generating a comprehensive national dataset spanning nearly a decade. This represents the largest single-country Euromelanoma analysis in Central–Eastern Europe and one of the most extensive datasets of its kind in the region. Data from 18,598 participants were evaluated to identify predictors of clinically suspicious skin cancers. The strongest risk factors were the presence of atypical (unusual) moles, older age, and a personal history of skin cancer. Heavy sunbed use emerged as a critical behavioral risk, particularly for melanoma. Screening motivation also influenced outcomes: individuals attending because of a changing lesion were more likely to have suspicious findings, whereas those attending for routine checks or because of family history were less likely. These findings underscore the importance of risk-stratified screening strategies, regulation of sunbed use, and targeted public health education—demonstrating how national Euromelanoma data can inform evidence-based screening policies and prevention efforts in Hungary and across Europe.

## 1. Introduction

Founded in Belgium in 1999 and led by dermatologists, Euromelanoma has evolved into a pan-European prevention initiative, now active in more than 30 countries [1,2,3]. The campaign aims to reduce the skin cancer burden by promoting primary and secondary prevention, raising public awareness of risk factors and warning signs, and encouraging early detection. Each year, participating countries adopt a unifying campaign theme, during which dermatologists provide free full-body skin examinations, and participants complete a standardized survey capturing demographics, phenotypic traits, and behavioral risk factors [3,4,5,6,7,8,9].

Euromelanoma has a threefold impact. Publicly, it implements coordinated communication strategies—including educational events, media campaigns, printed materials, and a multilingual online platform—to inform individuals about skin cancer prevention. Scientifically, standardized questionnaires and a central database enable dermatologists to collect cross-country data on skin cancer risk factors, enhancing predictive modeling and research. Policy-wise, the campaign provides evidence to guide public health strategies and to implement regulatory measures [1,2,3].

Hungary has actively participated in Euromelanoma since 2009, integrating nationwide awareness and screening activities into the campaign. These, alongside the introduction of novel systemic anticancer therapies, may have contributed to improved melanoma outcomes in the country; however, this relationship should be interpreted as associative rather than causal [10,11,12]. Between 2011 and 2015, melanoma incidence in Hungary rose by 16.4% in males and 18.82% in females, followed by stabilization or a slight decline through 2019, particularly among women (males: 12.77%, females: 11.35%) [11,12]. In 2011, 2426 new cases were identified, while in 2019, 2414 new cases were identified. During the 2011-2019 period, melanoma-specific mortality decreased by 16.6%, and age-standardized 5-year net survival increased from 90.6% (2011 to 2014) to 95.8% (2015–2019) [11,12].

Globally, melanoma incidence continues to rise, particularly among fair-skinned populations, making early detection and prevention an urgent public health priority [13]. Since the 2010s, the incidence of cutaneous melanoma has stabilized or decreased in some regions, such as Australia, New Zealand, and North America, although it continues to increase in Northern Europe [13]. Compared with Western Europe, Central–Eastern Europe remains relatively understudied in melanoma prevention research, partly due to regional differences in sun exposure patterns, tanning behaviors, healthcare access, and public awareness infrastructure [14].

Risk factors for melanoma and non-melanoma skin cancers (NMSC) have not been systematically evaluated in Hungary. Over the past decade, Euromelanoma screenings have offered a unique opportunity to investigate these predictors in a nationwide cohort. This effort produced the campaign’s largest single-country dataset, providing valuable insights from a Central–Eastern European perspective and supporting the development of risk-stratified prevention and screening strategies.

The present retrospective, cross-sectional study analyzes Hungarian Euromelanoma data from 2009 to 2018 with three aims: (i) to characterize participant demographics and screening motivations, (ii) to identify predictors of clinically suspicious melanoma and NMSC (basal cell and squamous cell carcinoma), and (iii) to contextualize these findings within European trends to inform tailored prevention and screening approaches.

## 2. Materials and Methods

Hungary’s Euromelanoma screenings were coordinated by the Hungarian Dermatological Society in collaboration with the Euromelanoma network. Public advertisements were disseminated via radio, television, newspapers, posters, and social media approximately one month prior to each campaign [15]. Although nationwide screenings began in 2007, standardized data collection under the Euromelanoma initiative started in 2009 and continued through 2018, before the interruption of in-person events due to the COVID-19 pandemic. This period was selected to provide a complete and methodologically consistent dataset for the analysis of national participation, risk factors, and screening outcomes. Screening examinations were conducted each May in publicly funded dermatology clinics, university and county hospitals, and private practices.

The standardized Euromelanoma questionnaire filled out during screening included 18 items—11 for participants and 7 for dermatologists—covering motivation for participation, demographics, phenotypic characteristics, behavioral risk factors, and clinical findings identified during full-body skin examination (Supplementary Material S1). The questionnaire was developed in 2008 by participating Euromelanoma countries under the coordination of epidemiologists and has since been used consistently across Europe [1]. National versions underwent forward-backward translation and layperson comprehension testing to ensure linguistic and conceptual equivalence (Supplementary Material S2).

Between 2009 and 2018, a total of 23,896 surveys were completed during Hungarian melanoma screening campaigns. Annual participation rates per 100,000 inhabitants are presented in Supplementary Table S3. After excluding surveys with missing or invalid data (primarily age and gender), 18,598 remained for the final analysis. Detailed steps of data filtering and exclusion criteria are provided in Supplementary Material S2. Descriptive statistics were generated using R version 4.2.3 within the RStudio environment.

Clinically relevant variables for multivariable analysis were selected based on prior literature and their potential predictive value. To ensure data quality, participants with invalid age values (e.g., age = 0 or ≥99) or missing age or gender were excluded. The analysis dataset included demographic variables (age, gender), established melanoma risk factors (e.g., history of sunburns, atypical nevi), personal and family history of skin cancer, and self-reported reasons for attending the screening.

Several variables were recoded or grouped to improve interpretability and statistical robustness. Skin type was derived from self-reported sun reactivity and classified into four predefined phototypes (I–IV), with an additional binary grouping (I/II vs. III/IV) used in the models, in accordance with previous Euromelanoma analyses [7]. Family history of melanoma was coded as a binary variable (“Yes” if any first-degree relatives were affected). Sunbed use was simplified to a binary variable (“Yes”/“No”), with heavy solarium use defined as >20 sessions per year, in line with previous multinational Euromelanoma analyses by Suppa et al. [16,17]. Sunny holidays were categorized based on duration (>2 weeks/year = “Yes”), and childhood sunburn before age 18 was recorded as a binary variable (“Yes”/“No”).

Nevus count was determined by the examining dermatologist and recorded in the standardized questionnaire (Supplementary Material S1). Participants were grouped into two categories: “<25” versus “≥25” nevi, in accordance with previous Euromelanoma analyses [7]. Clinical criteria for atypical nevi were predefined within the Euromelanoma questionnaire and included asymmetry, ill-defined borders, irregular pigmentation or color, and diameter > 6 mm, consistent with the ABCD framework. Nevi, lentigines, actinic keratoses, melanoma, and NMSC were diagnosed based on the dermatologist’s clinical and dermoscopic assessment. Clinically “suspicious” lesions referred to those judged as potentially malignant by the examining dermatologist. Each participant underwent a full-body skin examination, with dermoscopy performed in 92.4% of cases. Histopathological confirmation was not systematically available within the campaign; therefore, the reported outcomes reflect clinical suspicion rather than histologically verified diagnoses. The list and definition of all variables used in the Euromelanoma analysis dataset is available in Supplementary Table S4.

Variables with high rates of missing data were excluded to preserve statistical power and maximize sample size, a necessary trade-off given the relatively small number of participants with suspicious clinical findings (Supplementary Table S2). Three separate logistic regression models were constructed to identify independent predictors of: (i) any clinically suspicious skin cancer (melanoma, basal cell carcinoma (BCC), or squamous cell carcinoma (SCC)), (ii) suspicious melanoma, and (iii) suspicious NMSC (BCC and SCC) (Supplementary Material S2, Supplementary Table S2). All analyses were conducted using R version 4.2.3. More detailed descriptions of all supplementary materials accompanying the manuscript (Supplementary Description).

The study was approved by the National Council of Health Sciences, Scientific and Research Ethics Committee (certificate number: 32265-4/2015/EKU, approval period: 30 November 2015–30 November 2025) and the Regional and Institutional Review Board of Human Investigations at the University of Szeged (MEL-NAPOK-002, 3697/15).

## 3. Results

### 3.1. Participant Characteristics

**Figure 1:**
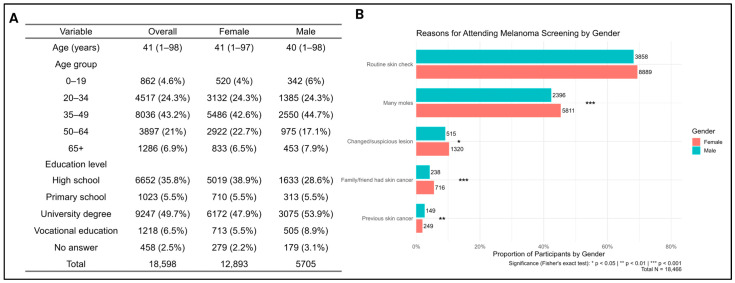
(**A**) Demographic characteristics of participants and (**B**) motivation for participation in the Euromelanoma screening campaign conducted in Hungary between 2009 and 2018. Of the 18,598 participants, 132 chose not to disclose their reason for attendance; the remaining 18,466 responses are shown.

A total of 18,598 surveys were analyzed. Participants’ ages ranged from 1 to 98 years (mean ± SD: 43.6 ± 16.4). Females accounted for 69.3% of respondents, and the predominant age group was 35–49 years (mean age: males 40, females 41) (Figure 1A). University-level education was reported by 53.9% of males and 47.9% of females.

Based on self-reported phototypes, types III (47.5%) and IV (29.5%) were most common (Table 1). A personal history of melanoma and NMSC was reported by 0.94% and 1.97% of participants, respectively, while 5.4% reported a family history of melanoma. In addition, 17.1% reported having ever used sunbeds, of whom 20.2% reported heavy use. One-third (34.7%) reported at least one severe sunburn before age 18. Always using sunscreen while sunbathing was reported by 51.7%, but only 19% did so during other outdoor activities lasting more than one hour.

Participation in previous skin cancer screenings, including earlier Euromelanoma events, was reported by 6075 (33.3%). The main reason for participation was routine skin checks (69.4% of women vs. 68.2% of men, *p* = 0.10), showing no significant gender difference (Figure 1B). However, women more frequently reported attending because of having many moles (45.4% vs. 42.3%, *p* < 0.001, OR 1.13, 95% CI 1.06–1.21), a suspicious or changed lesion (10.3% vs. 9.1%, *p* = 0.013; OR 1.15, 95% CI 1.03–1.28), or a family/friend history of skin cancer (5.6% vs. 4.2%, *p* < 0.001; OR 1.35, 95% CI 1.16–1.57). On the other hand, men more often reported a personal history of skin cancer (2.6% vs. 1.9%, *p* = 0.0035; OR 0.73, 95% CI 0.59–0.91).

Clinical examination revealed that 66.8% of participants had fewer than 25 nevi, and 30.7% had 25 or more (Table 2). Solar lentigines were observed in 61.1% of participants, atypical nevi in 22.6%, and actinic keratoses in 7.1%. Full-body skin examinations with dermoscopy were performed in 92.4% of participants.

### 3.2. Predictors of Clinically Suspicious Skin Cancers

**Figure 2:**
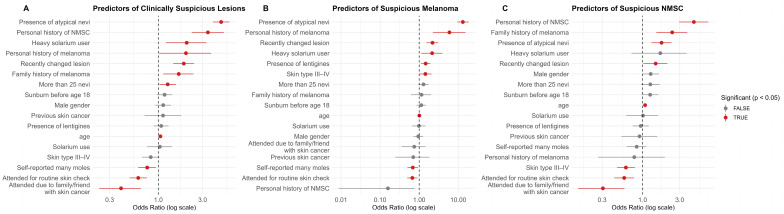
Logistic regression analyses of predictors for clinically suspicious (**A**) skin cancers (melanoma, BCC, SCC), (**B**) melanoma, and (**C**) non-melanoma skin cancers. Odds ratios shown on the figure present adjusted estimates. BCC: basal cell carcinoma, SCC: squamous cell carcinoma. The complete multivariable logistic regression outputs for all three clinical outcome models are available in Supplementary Table S1.

A total of 14,483 questionnaires were eligible to build a logistic regression model for predicting clinically suspicious skin cancers, and among them, 3.9% documented clinically suspicious skin cancers (melanoma, BCC, or SCC) (Supplementary Table S2). Strong predictors included atypical nevi (OR = 4.75, *p* < 0.001), personal history of NMSC (OR = 3.42) and melanoma (OR = 1.99), family history of melanoma (OR = 1.66, *p* = 0.008), >25 nevi (OR = 1.26, *p* = 0.026), heavy solarium use (OR = 2.02, *p* = 0.006), and older age (OR = 1.05 per year, *p* < 0.001) (Figure 2A). Motivation influenced findings: attending for a recently changed or suspicious lesion increased odds (OR = 1.88, *p* < 0.001), whereas attending for routine skin checks (OR = 0.61, *p* < 0.001), self-reporting “many moles” (OR = 0.76, *p* = 0.013), or having a family/friend history (OR = 0.40, *p* < 0.001) reduced odds. Gender, lentigines, sunscreen use, and history of sunburn were not significant.

### 3.3. Predictors of Clinically Suspicious Melanoma

A total of 14,473 surveys were eligible for inclusion in the logistic regression analysis to identify predictors of clinically suspicious melanoma, of which 1.7% documented clinically suspicious melanoma.

The strongest predictors were the presence of atypical nevi (OR = 13.12, *p* < 0.001) and a personal history of melanoma (OR = 5.95, *p* = 0.0003). Additional significant predictors included lentigines on the trunk (OR = 1.47, *p* = 0.006), heavy solarium use (OR = 2.15, *p* = 0.015), skin phototype III–IV (OR = 1.44, *p* = 0.039), and older age (OR = 1.01 per year, *p* = 0.022) (Figure 2B).

Screening motivation influenced detection: attending due to a recently changed or suspicious lesion markedly increased odds of melanoma suspicion (OR = 2.19, *p* < 0.001), whereas attending for a routine check (OR = 0.67, *p* = 0.007) or self-reporting “many moles” (OR = 0.67, *p* = 0.017) reduced odds. Older age slightly increased the risk (OR = 1.01, *p* = 0.022). Gender, family history of melanoma, personal history of NMSC, and history of sunburn before age 18 were not significantly associated, likely reflecting reduced statistical power due to the small number of suspicious melanoma cases.

### 3.4. Predictors of Clinically Suspicious NMSC

A total of 14,424 surveys were eligible for inclusion in the logistic regression model predicting clinically suspicious NMSC, of which 2.3% indicated suspicious NMSC (BCC and SCC).

Older age (OR = 1.08 per year, *p* < 0.0001) and a personal history of NMSC (OR = 4.75, *p* < 0.0001) were the strongest predictors (Figure 2C). Additional associations included the presence of atypical nevi (OR = 1.76, *p* < 0.001) and family history of melanoma (OR = 2.41, *p* < 0.001). In contrast, darker skin phototypes (III–IV) were associated with a lower risk compared to phototypes I and II (OR = 0.61, *p* < 0.001).

Screening motivation again influenced outcomes: participants attending because of a recently changed or suspicious lesion had increased odds (OR = 1.47, *p* = 0.034), whereas those attending for routine checks (OR = 0.58, *p* < 0.001) or due to family/friend history of skin cancer (OR = 0.31, *p* < 0.001) had decreased odds of NMSC suspicion. Family history was also associated with suspicious NMSC. Gender, sunbed use, early sunburn, and self-reported mole count were not significantly associated with NMSC risk.

## 4. Discussion

Hungary’s decade-long participation in Euromelanoma generated the largest single-country dataset within the campaign, offering the most comprehensive analysis to date from Central–Eastern Europe. Beyond confirming established clinical predictors, this study offered a unique opportunity to evaluate constitutional and behavioral risk factors as well as motivational determinants influencing screening outcomes—an aspect rarely examined in previous Euromelanoma analyses. It also provides valuable data on clinical predictors of skin cancer within a Central–Eastern European context.

Participants were predominantly women aged 35-49 years, highly educated, and mostly phototypes III and IV, in line with prior Euromelanoma reports [6,7,8,9,18,19]. The portion of clinically suspicious skin cancers detected was comparable to earlier studies (melanoma suspicion: 0.7–4.0%; BCC: 0–10.7%; SCC: 0–1.8%) [7,8].

Across all skin cancer types, atypical nevi, older age, and prior skin cancer were the strongest demographic and constitutional predictors. Age showed a particularly strong association with NMSC, reflecting the steep age-related rise in incidence, while its link with melanoma was weaker but consistent with global epidemiological patterns [20,21,22,23,24]. Atypical nevi were the most powerful predictor of melanoma, in agreement with prior literature [23,24,25,26]. They also modestly increased NMSC risk, likely reflecting shared phenotypic susceptibility and cumulative UV exposure rather than a causal relationship, underscoring their broader relevance beyond melanoma [27]. Lentigines on the trunk moderately increased melanoma suspicion, likely reflecting chronic sun exposure [23].

Pigmentation-related factors showed nuanced associations. Participants with phototypes III–IV were unexpectedly linked with higher melanoma suspicion, whereas fairer skin types (I–II) conferred higher NMSC risk. This contrasts with established evidence that melanoma risk typically increases with lighter skin tones, while the observed pattern for NMSC aligns with prior data [20,21,23]. These divergent findings may reflect differences in awareness, reporting or lesion presentation. In individuals with darker skin (especially phototype IV), melanoma may appear with subtler pigmentation, making early recognition more challenging. Additionally, subjective self-reporting of phototype and examiner-based lesion assessment may have contributed to this observation [28]. Although speculative, these findings highlight the need for awareness and diagnostic vigilance across phototypes and the importance of considering both biological susceptibility and behavioral context.

Personal history of melanoma or NMSC strongly increased risk across cancer types, underlining the central role of prior skin cancer in stratifying high-risk individuals [20,21,23]. While a personal history of melanoma remained a robust melanoma predictor, family history of melanoma was unexpectedly associated with NMSC—possibly reflecting shared UV exposure or overlapping genetic susceptibility, a relationship seldom reported in earlier studies [23,24,27,29,30,31,32]. A large prospective cohort study (>216,000 participants) found that a family history of melanoma conferred a 74% higher melanoma risk and modestly increased risks of SCC (HR 1.22) and BCC (HR 1.27), independent of pigmentary and environmental factors [30]. Genetic analyses further support shared susceptibility, as pigmentation-related variants near ASIP (encoding agouti signaling protein), TYR (encoding tyrosinase), and TYRP1 (encoding tyrosinase-related protein 1) were associated with both melanoma and BCC in European populations [31]. A case–control study of early-onset BCC identified family history of skin cancer as a strong independent risk factor (OR 2.4)—even after adjustment for pigment characteristics, UV exposure and MC1R genotype; the risk was highest—over threefold (OR 3.65, 95% CI 1.79–7.47)—among individuals with a family history of both melanoma and NMSC, particularly when a first-degree relative was diagnosed before age 50 [32]. Our finding that a family history of melanoma correlated with suspicious NMSC in the Hungarian cohort aligns with these reports, warranting further exploration.

Behavioral exposures were also critical. Based on earlier analyses within the Euromelanoma database, Hungary ranked among the top four European countries for sunbed use, with 17.1% of participants self-reporting ever-use versus 10.6% across Europe [1,16,17]. Self-reported heavy sunbed use (>20 sessions per year) was confirmed as an independent predictor for skin cancer overall and melanoma, reinforcing the well-documented hazards of artificial UV exposure [16,17,23]. These findings underscore the urgent need for regulatory and educational interventions targeting sunbed use in Hungary [7,17,33,34,35,36,37].

Motivation for screening was self-reported through multiple-choice responses. The main reason for participation was consistent with Euromelanoma trends [7,8,18,38]. Screening motivation shaped clinical yield. Participants presenting with a recently changed or suspicious lesion were more likely to have clinically relevant findings, while those attending for routine checks, “many moles”, or family/friend history were less likely. These results suggest that health-conscious individuals are not necessarily those at highest biological risk, underscoring the need for targeted public messaging. Educational efforts should prioritize recognition of changing or atypical lesions rather than general concern. Gender-specific differences also emerged: women were more likely to attend for mole counts or family/friend history, whereas men more often attended due to personal history. These findings suggest that, while general screening motivations are broadly similar, personal and familial risk factors drive gender-specific patterns in health-seeking behavior and may help guide future outreach strategies.

Taken together, these findings indicate that effective risk prediction requires integrating clinical signs (atypical nevi, lentigines), personal and family history, age, and high-risk UV behaviors, while also accounting for motivational factors. Consistent with reports from other countries, this multifactorial approach supports refined, risk-stratified screening and prevention strategies [2,4,19,39]. Building on the original Euromelanoma questionnaire, the European Skin Cancer Risk Factors Project (EUSCAP) developed a more detailed survey to assess risk factors with greater precision, offering future opportunities to refine predictive models and optimize prevention strategies across Europe [40].

## 5. Limitations

The relatively low number of clinically suspicious skin cancers limited statistical power, resulting in wide confidence intervals and possible underestimation of weaker associations. Several factors (e.g., gender, childhood sunburn, sunscreen use) showed no significant relationship, which may reflect a true lack of effect, limited statistical power, or measurement error. Reliance on self-reported variables such as mole count and phototype carries a risk of misclassification, and gender differences may further affect data reliability. Self-reported sunbed and sunscreen use may also be underreported, introducing bias.

Gender, family history of melanoma, personal history of NMSC, sunburn before age 18, and general sunbed use lost significance in the narrower melanoma models, likely reflecting limited power due to the small number of suspicious melanoma cases. Approximately one-third of participants reported prior skin checks, including earlier Euromelanoma screenings. As repeat attenders could not be identified or excluded, the exact number of unique participants remains unknown, potentially introducing clustering bias. Implementing longitudinal participant linkage in future campaigns could mitigate this limitation and enhance data precision.

Participants likely represented a more health-conscious subset of the general population, with less-educated or underserved groups underrepresented. This selection bias may limit generalizability and potentially underestimate true associations in the wider population. Future campaigns should therefore consider targeted outreach strategies—such as community-based events, simplified educational materials, tailored social media messaging, and engagement of primary care providers—to improve participation among lower-education groups. Further studies should also include cost-effectiveness analyses to assess the economic sustainability of Euromelanoma screening programs.

Finally, histopathological confirmation was inconsistently available, restricting interpretation to clinically suspicious rather than confirmed malignancies. This represents a major limitation, and future Euromelanoma campaigns should incorporate systematic biopsy follow-up for suspicious lesions to improve diagnostic accuracy and research validity.

## 6. Conclusions

This study represents the largest single-country Euromelanoma analysis in Central–Eastern Europe, providing over a decade of insights into population-level skin cancer risk factors in Hungary. Our models identified a blend of clinical signs, constitutional traits, and behavioral factors as key predictors of clinically suspicious skin cancers.

For melanoma, the strongest risk factors were atypical nevi, UV exposure behaviors—particularly heavy sunbed use—and a prior history of melanoma. For non-melanoma skin cancers, older age and a personal history of NMSC were dominant predictors. Across both cancer types, the presence of atypical nevi and any personal history of skin cancer consistently indicated elevated susceptibility, highlighting priority groups for targeted screening—notably older adults with atypical nevi and frequent solarium users.

Behavioral and motivational factors also influenced screening yield. Individuals attending for a changing or suspicious lesion were more likely to have relevant findings, underscoring the importance of tailored public messaging that emphasizes recognition of lesion change and atypical features rather than general concern or routine checks.

Heavy sunbed use remains a critical, modifiable risk factor for skin cancers—particularly melanoma—reinforcing the urgent need for strengthened regulation, health education, and public awareness initiatives in Hungary and similar European settings.

## Figures and Tables

**Table 1:** Summary of self-reported constitutional and behavioral risk factors of participants.

Constitutional Risk Factors		Overall	Females	Males
**Self-reported skin type**				
	My skin always burns, never tans My skin always burns, tans minimally or with difficulty My skin initially burns and then tans My skin burns minimally, tans readily	1037 (5.6%) 2962 (15.9%) 8831 (47.5%) 5486 (29.5%)	824 (6.4%) 2234 (17.3%) 5901 (45.8%) 3724 (28.9%)	213 (3.7%) 728 (12.8%) 2930 (51.4%) 1762 (30.9%)
**Family history of melanoma**				
	No Yes	17,421 (93.7%) 1015 (5.5%)	12,035 (93.3%) 734 (5.7%)	5386 (94.4%) 281 (4.9%)
**Personal history of melanoma**				
	No Yes	18,423 (99.1%) 175 (0.9%)	12,772 (99.1%) 121 (0.9%)	5651 (99.1%) 54 (0.9%)
**Personal history of NMSC**				
	No Yes	18,231 (98%) 367 (1.8%)	12,660 (98.2%) 233 (1.8%)	5571 (97.7%) 134 (2.3%)
**Behavioral Risk Factors**		**Overall**	**Females**	**Males**
**Sunburn before age 18 years (≥1 episode)**				
	No Yes	12,144 (65.3%) 6454 (34.7%)	8394 (65.1%) 4499 (34.9%)	3750 (65.7%) 1955 (34.3%)
**Sunny Holidays (>2 weeks/year)**				
	No Yes	11,494 (61.8%) 7104 (38.2%)	8339 (64.7%) 4554 (35.3%)	3155 (55.3%) 2550 (44.7%)
**Ever use solarium**				
	No Yes	14,268 (76.7%) 3179 (17.1%)	9442 (73.2%) 2710 (21%)	4826 (84.6%) 469 (8.2%)
**Sunscreen use when sunbathing**				
	Always Never Sometimes	9694 (52.1%) 1075 (5.8%) 4475 (24.1%)	7218 (56%) 601 (4.7%) 2847 (22.1%)	2476 (43.4%) 474 (8.3%) 1628 (28.5%)
**Sunscreen use when outdoors > 1 h (other than sunbathing)**				
	Always Never Sometimes	3539 (19%) 4321 (23.2%) 8569 (46.1%)	2748 (21.3%) 2741 (21.3%) 5843 (45.3%)	791 (13.9%) 1580 (27.7%) 2726 (47.8%)
**≥1 year in countries with high sun before age 18**				
	No Yes	18,422 (99.1%) 176 (0.9%)	12,773 (99.1%) 120 (0.9%)	5649 (99%) 56 (1%)
**≥1 year in countries with high sun before age 18**				
	No Yes	18,044 (97%) 554 (3%)	12,510 (97%) 383 (3%)	5534 (97%) 171 (3%)

**Table 2:** Summary of clinical findings identified during full-body dermatological examinations.

Clinical Findings	Levels	Overall	Females	Males
**Total nevus count**				
	<25 >100 25–50 50–100	12,164 (66.8%) 469 (2.6%) 2820 (22.3%) 1400 (7.7%)	8649 (68.5%) 276 (2.2%) 2820 (22.3%) 877 (6.9%)	3515 (62.9%) 193 (3.5%) 1361 (24.3%) 523 (9.4%)
**Total nevus count (grouped)**				
	<25 >25	12,164 (66.8%) 6050 (33.2%)	8649 (68.5%) 3973 (31.5%)	3515 (62.9%) 2077 (37.1%)
**Presence of atypical nevi**				
	No Yes	13,460 (77.4%) 3934 (22.6%)	9461 (78.5%) 2590 (21.5%)	3999 (74.8%) 1344 (25.2%)
**Presence of lentigines**				
	No Yes	11,193 (63.1%) 6542 (36.9%)	7812 (63.6%) 4480 (36.4%)	3381 (62.1%) 2062 (37.9%)
**Presence of AK**				
	No Yes	16,323 (92.9%) 1244 (7.1%)	11,359 (93.1%) 841 (6.9%)	4964 (92.5%) 403 (7.5%)
**Individuals screened with suspected skin cancer of any type**				
	No Yes	16,186 (95.7%) 725 (4.3%)	11,180 (96%) 469 (4%)	5006 (95.1%) 256 (4.9%)
**Individuals screened with suspected melanoma**				
	No Yes	16,575 (98.2%) 311 (1.8%)	11,426 (98.2%) 206 (1.8%)	5149 (98%) 105 (2%)
**Individuals screened with suspected BCC**				
	No Yes	16,423 (97.6%) 404 (2.4%)	11,338 (97.8%) 256 (2.2%)	5085 (97.2%) 148 (2.8%)
**Individuals screened with suspected SCC**				
	No Yes	16,711 (99.7%) 57 (0.3%)	11,515 (99.7%) 38 (0.3%)	5196 (99.6%) 19 (0.4%)

## Data Availability

The Euromelanoma dataset cannot be made publicly available due to privacy, legal, and ethical considerations, but data are available upon request to the Euromelanoma consortium.

## Supplementary Materials

The following supporting information can be downloaded at: https://www.mdpi.com/article/10.3390/cancers17233749/s1, Supplementary Description: detailed descriptions of all supplementary materials accompanying the manuscript, Material S1: Euromelanoma questionnaire, Material S2: Filtering Steps, Table S1: Model summaries, Table S2: Variable missingness, Table S3: Participation rates, Table S4: Variable descriptions.
