# Supplementary material for: Ten Years of Euromelanoma in Hungary: Nationwide Trends and Risk Factors for Skin Cancer in Central–Eastern Europe
Source: Cancers (Basel). 2025 Nov 24;17(23):3749. doi: 10.3390/cancers17233749 (PMC12691246; doi:10.3390/cancers17233749)
Supplement: Supplementary file 1 [file cancers-17-03749-s001.zip › supplementary_description.pdf]

Supplementary Description

This document provides detailed descriptions of all supplementary materials accompanying the manuscript. Each supplementary item is uploaded as a separate file under its final name. All supplementary material available at the publications GitHub repository.

GitHub repo: https://github.com/gunyorka/euromelanoma-hungary/

Supplementary Table S1. (Table S1):

Description:

This Excel file contains the complete multivariable logistic regression outputs for all three clinical outcome models used in the Euromelanoma analysis. Each worksheet represents one model and lists the adjusted odds ratios (aORs), 95% confidence intervals, and p-values for all predictors included.

Worksheets:

1. Suspicious_melanoma – Logistic regression model predicting the likelihood of a lesion being clinically suspicious for melanoma.
2. NMSC – Model predicting clinical suspicion of non-melanoma skin cancer (NMSC), combining basal cell carcinoma (BCC) and squamous cell carcinoma (SCC) cases to preserve statistical power.
3. Melanoma_plus_NMSC – Model including all clinically suspicious lesions (melanoma or NMSC) under the composite variable suspected_all.

Variable definitions:
Predictors include demographic (age, gender), phenotypic (phototype, nevus count, presence of atypical moles), and behavioral (sunburn history, sunscreen use, solarium exposure, travel to sunny countries) factors. Each coefficient represents the adjusted effect of the variable on clinical suspicion, controlling for all other covariates. For detailed description see Supplementary Table S4.

Supplementary Table S2. (Table S2):

Description:

This table summarizes missing data patterns for all variables included in the Euromelanoma analysis dataset (n = 18,598). For each variable, the table reports:

- Overall missingness: Number and percentage of missing observations across the entire dataset.
- Conditional missingness: Missing counts and percentages within participants who were clinically classified as
    - suspected_all = "Yes" (any clinically suspicious lesion),
    - suspected_melanoma = "Yes", and

    - suspected_NMSC = "Yes".
- Total "Yes" counts for each outcome variable (providing denominators for the conditional missingness columns).

Variables are ordered by decreasing overall missingness to illustrate which factors most contributed to data reduction during complete-case modeling. This table therefore explains the differences in sample sizes between descriptive and regression analyses, showing which predictors were excluded due to high missingness or non-response.

Supplementary Table S3 (Table S3):

Description:
This table summarizes annual participation rates in the Hungarian Euromelanoma campaign, normalized to the national population size. For each campaign year (2009–2018), the table lists:

- Number of screening participants (survey responses collected),
- Estimated Hungarian mid-year population (in millions), and
- Calculated participation rate per 100,000 inhabitants.

Population figures were obtained from the Hungarian Central Statistical Office (KSH). These rates quantify the yearly coverage of the Euromelanoma screening program and illustrate temporal trends in national campaign reach.

Supplementary Table S4 (Table S4)

Description:
This table lists and defines all variables used in the Euromelanoma analysis dataset (n = 18,598). It includes 36 variables covering demographic, phenotypic, behavioral, and clinical factors, as well as derived variables created during data preprocessing in R. Each entry provides a short, clear explanation of the variable's meaning and role in the analysis. The table allows readers to understand the origin and interpretation of each covariate used in the logistic regression models and descriptive statistics, ensuring full transparency and reproducibility of the dataset.

Supplementary Material S1

The Euromelanoma questionnaire was developed in 2008 through collaboration among participating Euromelanoma countries, coordinated by epidemiologists Jean-Luc Bulliard and Esther de Vries (*Stratigos et al.*, *Br J Dermatol.*, 2012). Since then, it has been applied consistently in more than 30 European countries to gather harmonized data on demographic, phenotypic, behavioral, and clinical factors related to skin cancer. Each national version underwent forward- and backward-translation and comprehension testing among laypersons to ensure linguistic and conceptual equivalence. Although no formal psychometric validation was performed, this pragmatic validation process ensured comparability across countries. Data collected with this instrument have supported numerous peer-reviewed publications, including large multinational Euromelanoma analyses.

Supplementary Material S2

This document outlines the complete data-cleaning and filtering workflow applied to the raw Euromelanoma questionnaire dataset (n = 23 896). It describes each preprocessing step — from variable renaming and age reconstruction to exclusion of records with invalid or missing values — and presents a transparent overview of how the number of clinically suspicious melanoma and non-melanoma skin cancer (NMSC) cases changed throughout data preparation.

Supplementary Figure S1. (Figure S1):

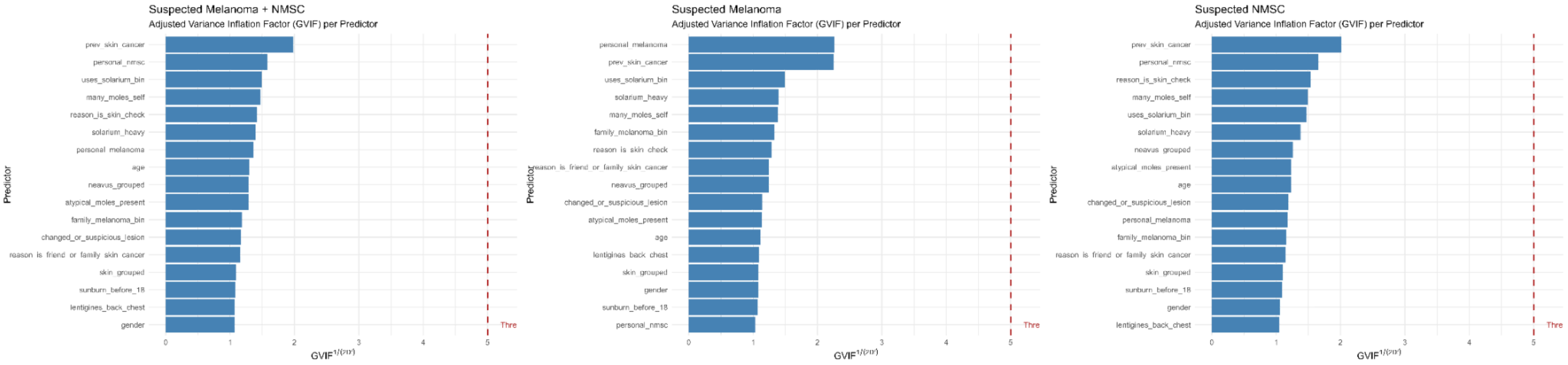

Figure S1. Variance Inflation Factor (VIF) assessment of multicollinearity among predictors in the three logistic regression models. Adjusted GVIF^(1/(2·Df)) values for all predictors were well below the conventional threshold of 5, indicating no concerning multicollinearity. Most variables clustered between 1.0 and 2.0, suggesting low correlation among predictors. The highest values (approximately 2.0 – 2.3) were observed for prev_skin_cancer and personal_nmsc, which are biologically related but still remained well below problematic levels. No variables approached the critical cutoff (red dashed line at 5), confirming that multicollinearity is not an issue in any of the models.

High resolution figure available at the publications GitHub repository: https://github.com/gunyorka/euromelanoma-hungary/blob/main/plots/vif_plot_highres.png
