# Supplementary material for: Ten Years of Euromelanoma in Hungary: Nationwide Trends and Risk Factors for Skin Cancer in Central–Eastern Europe
Source: Cancers (Basel). 2025 Nov 24;17(23):3749. doi: 10.3390/cancers17233749 (PMC12691246; doi:10.3390/cancers17233749)
Supplement: Supplementary file 1 [file cancers-17-03749-s001.zip › Supplementary_Material_S1_euromelanoma_questionairre.pdf]

# EUROMELANOMA QUESTIONNAIRE 2012

*To be completed by person screened:*

**1. Gender:** Male ☐ Female ☐

**2. Date of Birth:** (day/month/year) ☐☐/☐☐/☐☐☐☐

**3. What is Your highest degree of education?**

☐ Primary school ☐ High school ☐ Vocational education ☐ University degree

**4. Why did you participate in Euromelanoma? (Tick all that apply)**

☐ I have many moles
☐ Recently changed or suspicious lesion
☐ I was previously diagnosed with a skin cancer
☐ I have a family member or friend with skin cancer
☐ Because I want to have my skin checked

**5. Have you previously received a full skin examination? (including Euromelanoma)**

No ☐ Yes ☐ __ Number of times

**6. Did or do you have an outdoor occupation? If yes, for how many years?**

☐ No
☐ Yes, for: ☐ 1 year or less
☐ more than 1, until 5 years
☐ more than 5, until 10 years
☐ more than 10 years

**7. How does your skin react to the summer sun?**

☐ My skin always burns, never tans
☐ My skin always burns, tans minimally or with difficulty
☐ My skin initially burns and then tans
☐ My skin burns minimally, tans readily

**8. Did you suffer from severe sunburn (a painful sunburn, with intense redness or blistering, lasting for 2 days or more) before the age of 18?**

☐ No ☐ Yes ☐I don't remember

## 9. How often do you use sunscreens when you are exposed to the sun?

9.1. When you are outdoor for > 1 hour (other than sunbathing):

☐ Never ☐ Sometimes ☐ Always

9.2. Do you apply sunscreen when you are sunbathing:

☐ Never ☐ Sometimes ☐ Always

9.3. I never take a sunbath: ☐

## 10. Did you spend in total one year or more in a country with much higher sun exposure than the country where you currently live?

☐ No

☐ Yes, before the age of 18: ☐☐ years

☐ Yes, after the age of 18: ☐☐ years

## 11. Sun exposure during adulthood?

11.1. Number of weeks per year at sunny holidays:

☐ 0 ☐ 2 weeks or less ☐ more than 2 weeks

11.2. Do you use solarium?

☐ No ☐ Yes ≤ 20 sessions or less/year ☐ Yes, 21 or more sessions/ year

11.3. Number of years using solarium (including in the past only): ☐☐ yrs

*To be completed by physician:*

**12. Family history of melanoma** (melanoma in first degree relatives: father, mother, brother and sister)**:**

☐ No ☐ Yes:1 relative ☐ Yes: ≥2 first degree relatives ☐ Patient doesn't know

**13. Personal history of skin cancer:**

☐ No

☐ Yes, melanoma

☐ Yes, non-melanoma skin cancer

☐ Patient doesn't know if he/she has had skin cancer

**14. Skin examination performed today:** ☐ full ☐ partial

**15. I used dermoscopy to examine this patient:** ☐ Yes ☐ No

**16. Clinical examination:**

16.1. Number of moles: ☐ <25 ☐ 25-50 ☐ 50-100 ☐ >100

16.2 Presence of lentigines on the back / chest: ☐ No ☐ Yes

16.3 Presence of atypical moles (according to definition*):

☐ No ☐ Yes Number: ___

** (asymmetry, ill-defined border, irregular pigmentation/color, diameter >6mm)*

16.4 Presence of actinic keratoses: ☐ No ☐ Yes

**17. Clinically suspicious lesions[#]:**

1. Melanoma: ☐ No * ☐Yes __ Number

2. BCC: ☐ No + ☐Yes __ Number

3. SCC: ☐ No # ☐Yes __ Number

4. Other or clinically undefined: ☐ No ☐Yes __ Number

**18. The lesion was first detected by (please fill only when there is a clinically suspicious lesion observed by the dermatologist, if there are several lesions, the clinically most important one):**

☐ patient ☐ dermatologist ☐ another health professional ☐ spouse/partner ☐ other person
