# Supplementary material for: Ten Years of Euromelanoma in Hungary: Nationwide Trends and Risk Factors for Skin Cancer in Central–Eastern Europe
Source: Cancers (Basel). 2025 Nov 24;17(23):3749. doi: 10.3390/cancers17233749 (PMC12691246; doi:10.3390/cancers17233749)
Supplement: Supplementary file 1 [file cancers-17-03749-s001.zip › Supplementary_Material_S2_filtering_steps.pdf]

Summary of Case Counts Across Analysis Steps

To ensure transparency, the following overview clarifies how the number of **clinically suspicious lesions** changed across data processing and analysis stages:

1. **Raw dataset**
   - Total survey responses: **23,896**
   - Clinically suspicious lesions reported by dermatologists:
     - **Suspicious melanoma:** 501
     - **Suspicious squamous cell carcinoma (SCC):** 65
     - **Suspicious basal cell carcinoma (BCC):** 521

2. **Data cleaning and filtering process**
   - **Variable standardization and renaming**

     All raw columns were renamed to descriptive English identifiers corresponding to the original Euromelanoma questionnaire items (e.g., many_moles_self, changed_or_suspicious_lesion, sun_reaction, used_dermoscopy, suspected_melanoma, suspected_bcc, suspected_scc).

   - **Age reconstruction and validation**
     - **i.** Birth-year information was extracted from two partially overlapping variables (`birth_day_1`, `birth_year`).
     - **ii.** A unified birth year (`birth_year_combined`) was calculated, and age was computed as
       age = euromelanoma_year – birth_year_combined.
     - **iii.** An age-group factor (0–19, 20–34, 35–49, 50–64, 65+) was derived for descriptive purposes.
   - **Unique participant identifiers**
     A unique ID (`eumel_id`) was generated by concatenating the original record ID and screening year (e.g., *EUMEL_1234_2012*).

   - **Data type harmonization**
     - **i.** Empty strings (`""`) and placeholder values (`"9999"`) were recoded as missing (`NA`).
     - ii. Categorical variables were converted to factors, and numeric variables to numeric type.

iii. Responses coded as `"0"` for *uses_solarium* were converted to `"No"` for consistency.

- **Filtering criteria**
  The cleaned dataset (`mel_df`) was restricted to valid, interpretable records by applying the following filters:
  i. Entries were removed if Age variable = 0 or > 100 (possible recoding mistakes)
  ii. Non-missing gender and age values.

After filtering, 18 598 responses remained for descriptive and modeling analyses.

3. **Suspicious lesion count after filtering (n = 18,598)**
   - **Suspicious melanoma:** 311
   - **Suspicious SCC:** 57
   - **Suspicious BCC:** 404

4. **Creation of pooled non-melanoma category (NMSC)**

   - To maintain statistical power, suspicious SCC and BCC were combined into a single variable, **"suspicious NMSC."**
   - The resulting count of **434 suspicious NMSC cases** is slightly lower than the simple sum of 57 + 404 = 461, because only records with **non-missing values for both SCC and BCC fields** were retained.

5. **Derived "any suspicious lesion" variable (suspected_all)**

   - A combined indicator (**suspected_all**) was created to represent participants with *any* suspicious lesion (melanoma or NMSC).
   - After applying complete-case filtering for model covariates, this dataset included **14,483 participants**, of whom **561** had a clinically suspicious lesion.

6. **Final logistic regression datasets**

   - **Suspicious melanoma model:** 14,473 participants; **242 cases**.
   - **Suspicious NMSC model:** 14,424 participants; **331 cases**.

    - **Any suspicious lesion model:** 14,483 participants; **561 cases**.

Interpretation

- The stepwise decrease in total counts reflects progressive filtering for completeness of key variables (age, gender, and covariates) required for regression.
- Pooling SCC and BCC as NMSC followed the standard Euromelanoma convention to preserve statistical power given low subtype frequencies.
- The final model-specific case counts thus represent complete-case datasets, optimized for comparability and valid multivariable inference.
